# Specific refolding pathway of viscumin A chain in membrane-like medium **reveals a possible mechanism of toxin entry into cell**

**DOI:** 10.1038/s41598-018-36310-6

**Published:** 2019-01-23

**Authors:** Pavel E. Volynsky, Dmitry E. Nolde, Galina S. Zakharova, Rex A. Palmer, Alexander G. Tonevitsky, Roman G. Efremov

**Affiliations:** 1 M.M. Shemyakin & Yu.A. Ovchinnikov Institute of Bioorganic Chemistry, Russian Academy of Sciences, Miklukho-Maklaya Street, 16/10, Moscow, 117997 Russia; 2 Scientific and Research Center “BioClinicum”, Ugreshkaya Street, 2/85, Moscow, 115088 Russia; 3 Department of Crystallography, Biochemical Sciences, Birkbeck College, Malet St, London, WC1E7HX UK; 4 National Research University Higher School of Economics, Myasnitskaya ul. 20, 101000 Moscow, Russia; 5 Moscow Institute of Physics and Technology, Institutsky per., 9, 141700 Dolgoprudnyi, Russia

## Abstract

How is a water-soluble globular protein able to spontaneously cross a cellular membrane? It is commonly accepted that it undergoes significant structural rearrangements on the lipid-water interface, thus acquiring membrane binding and penetration ability. In this study molecular dynamics (MD) simulations have been used to explore large-scale conformational changes of the globular viscumin A chain in a complex environment – comprising urea and chloroform/methanol (CHCl_3_/MeOH) mixture. Being well-packed in aqueous solution, viscumin A undergoes global structural rearrangements in both organic media. In urea, the protein is “swelling” and gradually loses its long-distance contacts, thus resembling the “molten globule” state. In CHCl_3_/MeOH, viscumin A is in effect turned “inside out”. This is accompanied with strengthening of the secondary structure and surface exposure of hydrophobic epitopes originally buried inside the globule. Resulting solvent-adapted models were further subjected to Monte Carlo simulations with an implicit hydrophobic slab membrane. In contrast to only a few point surface contacts in water and two short regions with weak protein-lipid interactions in urea, MD-derived structures in CHCl_3_/MeOH reveal multiple determinants of membrane interaction. Consequently it is now possible to propose a specific pathway for the structural adaptation of viscumin A with respect to the cell membrane – a probable first step of its translocation into cytoplasmic targets.

## Introduction

The functioning of many proteins that possess a well-defined and densely packed spatial structure in water is often associated with their trafficking into and out of the cell *via* crossing the lipid membrane. Apart from proteins that overcome such a barrier with the help of different assisting systems (for example translocon, membrane transporters and carriers), other proteins have been shown capable of spontaneous insertion into the hydrophobic membrane milieu with subsequent translocation and refolding on the opposite side of the lipid bilayer^1^. Accommodation in the lipid environment requires serious reorganization of the protein structure. This is because some important characteristics of membrane proteins are considered to be “inverted” as compared to globular proteins^1–3^. For instance, strong hydrophobic moieties have to appear on the external surface, while most of the polar/charged residues are buried inside. It is evident that in most of the cases, such a transition is accompanied with global conformational changes which trigger the initial interaction of the water-soluble protein with the membrane protein.

The problem of protein structural rearrangements induced by membranes has been extensively studied employing both experimental and computational methods^3^. As a result, it is well accepted now that the membrane-induced global or partial protein transformation (often, the term “denaturation” is used as well) is mainly caused by the negative electrostatic potential of the membrane surface^4^ and/or low dielectric permeability of lipid bilayers with respect to aqueous solution^1,5^. In experiments, membrane effects are often imitated with water-alcohol mixtures – to study the joint action of the local decrease of pH and dielectric permeability^3^. It was shown that in such environments many ordered proteins lose their native spatial structure and adopt molten globule-like intermediate states. Typically, this is accompanied by the increase in their α-helical content. Despite a large body of experimental observations made with different techniques including CD, fluorescence and calorimetry, detailed atomic-scale mechanisms of such protein rearrangements are not well understood because the required high-resolution data (X-ray and NMR) are still very difficult or even impossible to obtain. This, in turn, is caused by the fact that the proteins undergoing conformational transitions in membrane-like environment represent a complex and heterogeneous ensemble of unsynchronized molecules, thus hampering occurrence of some “average” states with a defined 3D structure.

With respect to such situations a precise picture can be obtained with the help of atomistic simulations. The most adequate approach lies in studies of conformational transitions of an initially ordered water-soluble protein emerging from aqueous solution on the membrane surface. Although protein-membrane interactions have been extensively studied in computational experiments^6,7^, such works either considered just initial stages of protein adsorption on membrane or protein insertion and translocation were accelerated using for example steered dynamics^8^, special restraints^9^ or simplified models including for example coarse-grained ones^6^. This is because the global protein structural reorganization in the presence of explicit lipid bilayer and water is a slow process whose atomistic description on proper time scales is too computationally demanding and cannot be reached within a realistic timescale. On the other hand, application of simplified models, like implicit or coarse-grained presentations of a protein and a membrane, is limited because of difficulties in the description of microscopic interactions (especially H-bonds) and atomic-scale conformational dynamics of the systems under study. In addition, the role of the membrane (the so-called “membrane response”)^10^ is very important since protein-membrane interactions critically depend on mutual adaptation of both players^11^.

One alternative solution to the problem lies in atomistic simulations of initially ordered protein in environments with different physico-chemical properties realistically mimicking cell membranes of various types. For instance, the membrane effects can be modelled by organic solvents, in particular – the chloroform/methanol mixture. This solvent has been widely and successfully used in NMR studies of membrane proteins and their transmembrane domains^12,13^. To answer the aforementioned questions concerning the most probable pathways of protein transition from globular to membrane-bound forms, the following issues have to be explored: (i) Whether structural rearrangements in membrane mimics are reproducible in independent long-term MD simulations; (ii) Whether such structural changes differ from those obtained in other denaturing conditions, which are not related to the effects of membranes. In this case simulations in another – “non-membrane like” – denaturing solvent are required for example in urea. Also the thermal denaturation pathway can be checked *via* simulations at elevated temperature. One more question remains: (iii) Are the resulting transformed conformations better suited to interaction with a model membrane than the globular state and/or the “non-membrane” denatured states? To check this and to delineate potential membrane-binding sites, the aforementioned protein models were further subjected to Monte Carlo simulations with an implicit hydrophobic slab membrane. Based on the totality of the computational results, we propose (at least in a first approximation) a molecular mechanism of the membrane-induced reorganization and translocation of a water-soluble ordered protein.

In the present study, we employed this computational strategy to delineate a putative way of trafficking into the cell of the A chain of mistletoe lectin I (MLI) from the plant *Viscum album*. MLI (or viscumin) is a toxic lectin, which consists of two subunits (A and B) linked by a disulfide bond^14^. While the lectin activity and specificity determinants are located in the B-chain, the A-chain has rRNA N-glycosidase activity and irreversibly inhibits protein biosynthesis by cleavage of the conserved GAGA loop of 28S rRNA^15^. That is why MLI is classified as a type II ribosome-inactivating protein (RIP). Structural organization and functioning of MLI are very similar to those in another type II RIP – ricin.

Binding of the B chain lectin domains to carbohydrate moieties exposed on the cell surface triggers receptor-mediated endocytosis of viscumin holotoxin and further trafficking of MLI to the trans-Golgi network and endoplasmic reticulum^16^. After interchain Cys-Cys bond reduction, the A-chain is translocated across the membrane into the cytosol^17,18^ and reaches its ribosomal target^19^.

For many years it was assumed that the membrane-induced toxin unfolding facilitating subsequent translocation is assisted by other receptor systems. However it was recently shown that the A chain of ricin (RTA) can undergo spontaneous structural reorganization in the presence of membrane^20^. Prior to translocation into the cytosol, unfolding of the viscumin A-chain occurs^21^ and this also happens to the ricin A-chain^22^. In addition it has been shown that RTA can directly interact with lipid membranes of different composition. For instance, after Triton X-114 extraction RTA (not the holotoxin) is present in the detergent phase^23^. In 2009, binding of RTA to Endoplasmic reticulum (ER) microsomal membranes and to negatively charged liposomes was demonstrated using gel-filtration chromatography, fluorescent labeling and CD^20^. Therefore, binding to a membrane surface is an intrinsic property of RTA and, most likely similarly to the MLI A chain (MLA).

These considerations strongly suggest that computational MD/MC-experiments on toxin rearrangement in membrane-mimicking solvents is a highly promising prospect since they can help in delineation of the molecular aspects of the early stages of protein translocation. This, in turn, opens new avenues for rational selection of chemical and bioengineering tools capable of modulating the membrane passage of the catalytic subunits of RIPs and other proteins thus opening up new avenues for the prediction of properties of RIP-containing immunotoxins^24^.

## Results

### Overall flowchart of the study

Initially large-scale conformational rearrangements of MLA were explored in two explicit organic solvents – CHCl_3_/MeOH mixture and urea, as well as in water. This was done using atomistic MD simulations at an elevated temperature (340 K) - in order to accelerate conformational sampling although still remaining under non-denaturating conditions. For control purposes, one MD run was also performed in water at normal temperature (310 K). The solvents were chosen to mimic a water-membrane interface and a standard denaturing environment, respectively. The starting structure used for MD was in all cases the high-resolution X-ray spatial model of the protein. As a result, four sets of MD trajectories of MLA in different environments were accumulated and analyzed. Structural features of the protein were evaluated including: overall compactness of the structure (gyration radius, residue-residue contact maps); secondary structure, root-mean-square deviation (RMSD) from the starting model; time evolution and location of the most prominent conformational transitions and structurally rigid/flexible regions; surface characteristics (total area, hydrophobic/hydrophilic regions), overall 3D_1D score and its distribution along the sequence, and time-dependent location of hydrophobic clusters on the protein surface. One of the main problems considered here, is the large-scale dynamic rearrangement of the protein spatial structure. A suitable parameter for delineation of unstable segments in a protein is the root-mean-square fluctuation (RMSF) of atomic coordinates. However, it is inappropriate in the case of large structural changes, thus calling for more sophisticated analysis. To this end, we employed the “fluctuation maps” (see Methods). The principal aims of this MD study were the following: (i) To find a putative pathway for MLA structural reorganization resembling that occurring near the membrane interface; (ii) To compare such a “membrane-induced” path with the denaturation of MLA usually observed in organic solvents (eg urea) or with respect to elevated temperature and to test specificity of the former transition.

At the second stage, the resulting MD-states obtained in CHCl_3_/MeOH and urea, along with the two structures in water (at 340 K and 310 K), were explored with respect to their ability to interact with the membrane interface. This was accomplished with Monte Carlo (MC) simulations in the presence of the “hydrophobic slab” membrane model. In this case, the main parameter to be varied was the orientation of MLA with respect to the membrane surface, the protein spatial structure being effectively preserved. This means that such a computational experiment simply explores the conformational ability of a rigid protein model (after its reorganization in MD) to adapt to the heterogeneous water-membrane interface. These simulations do not attempt to explore global structural rearrangements of MLA on membrane adsorption and embedding. Instead, the objectives here were the following: (i) To check, whether some of these states (especially those obtained in the CHCl_3_/MeOH mixture) are capable of interacting with the membrane or not; (ii) To identify (if any) new hydrophobic determinants appearing on the protein surface upon protein adaptation to the membrane environment; (iii) To compare computational results with available experimental data on the interactions of RIPs with membranes. Possible membrane binding sites determined from such considerations are of primary importance because they promote an understanding of the atomistic grounds of the membrane-induced adaptation of MLA to the cellular membrane – the first step in its spontaneous translocation into the cytoplasm.

### Solvent-dependent evolution of MLA structure as probed by MD simulations

**Figure 1:**
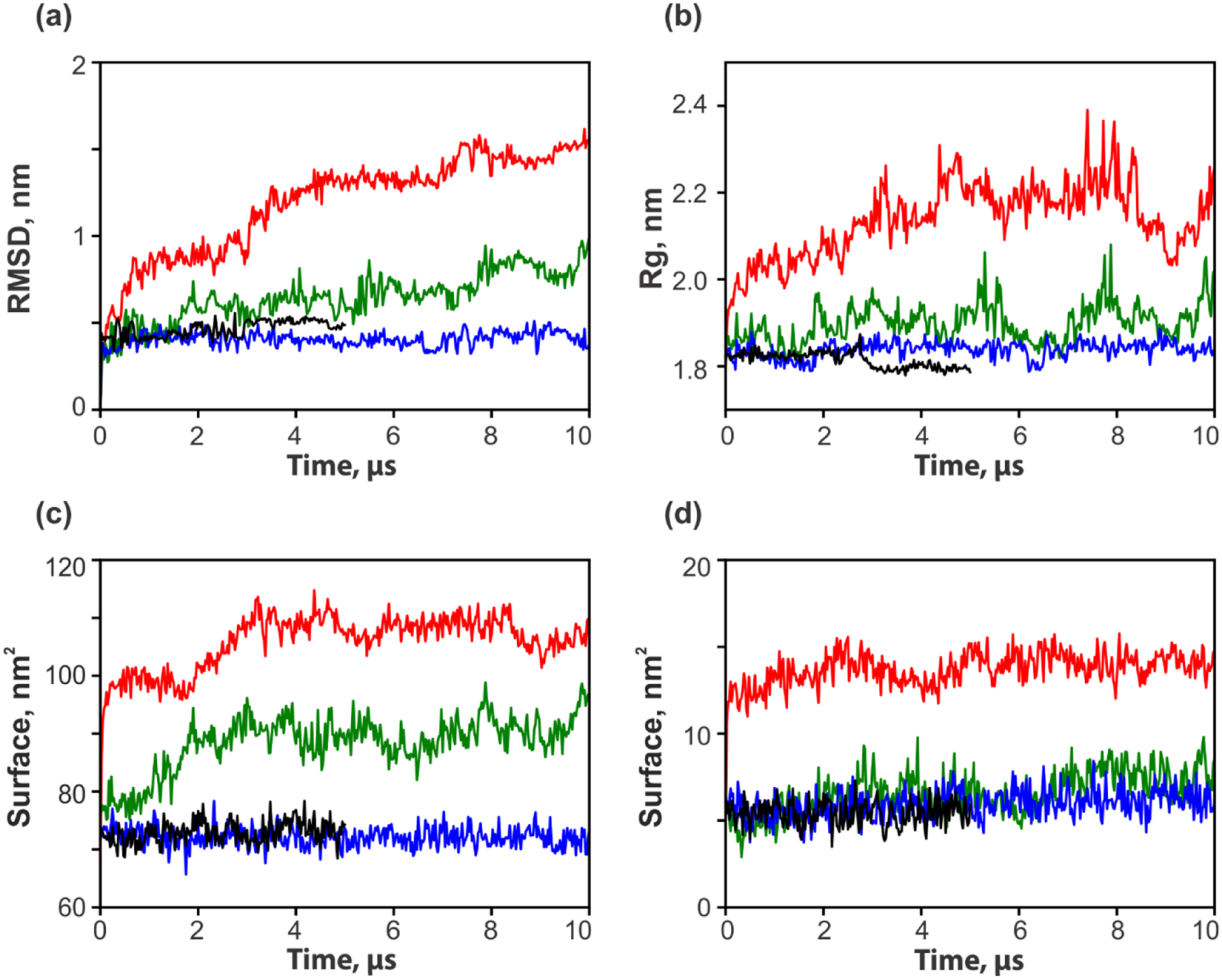
Evolution of overall protein properties in the course of MD simulations. (**a**) RMSD from the initial structure. (**b**) Radius of gyration. (**c**) Accessible surface area of the protein. (**d**) Hydrophobic accessible surface area of the protein. Black, blue, green, and red curves correspond to MD simulations in water (310 and 340 K), urea and CHCl_3_/MeOH mixture, respectively.

Results of MD simulations illustrate that the behavior of viscumin A critically depends on the solvent employed. Global characteristics of the protein - gyration radius, RMSD from the starting model, total and hydrophobic surface area – in different environments are presented in Fig. 1. It is seen that the protein structure in water at 310 K and at 340 K was stable: corresponding RMSDs from the starting model were less than 0.5 nm (Fig. 1a). In addition, at 310 K the protein became even more compact during MD – its radius of gyration decreased from 1.83 to 1.80 nm (Fig. 1b), while the total and the hydrophobic surface areas remain unchanged (Fig. 1c,d). It is worth noting that the overall stability of the MLA structure in aqueous solution at normal temperature (310 K) agrees well with the experimental data – MLA remains stable under such conditions. This proves that the MD simulation parameters were appropriate and hence, can be applied to modeling of the protein in other environments. We should also note that the spatial structure in water (after 5-μs MD at 310 K) reveals very good quality assessed using the 3D_1D technique by D. Eisenberg *et al*.^25^: the total value of the score (*S*) is ≈109, whereas that for the “ideally packed” 254-residue protein is ≈113. As seen in Figure S1, the water-adapted model of MLA includes very few sequence regions with negative local 3D-1D score (*S*_*i*_). This means that the globular structure of MLA is well-packed and adapted to the aqueous environment. Increasing the temperature to 340 K leads to some structural destabilization, which is reflected in the corresponding 3D_1D plot (Fig. S2, red curve). As follows from the analysis of differential plots between the curves obtained at 310 and 340 K, upon heating, the most prominent changes occur in the sequence regions 25–36, 226–235 and 240–247. Such potentially “weak points” of the model at 340 K include the inter-subunit interface, which is mainly formed by hydrophobic residues. Upon dissociation of the A and B subunits, these surface zones become exposed to water. In addition elevation of the temperature perturbs these regions (see below for further details) and does not lead to global protein destabilization. By contrast, in less polar organic solvents (like urea and CHCl_3_/MeOH), such regions play important roles in the structural rearrangements of the MLA 3D structure (see below).

As shown in Fig. 1 MLA is less stable in urea than it is in water: the protein structure is “swelling” in the course of MD. Thus the RMSD and radius of gyration increase to 0.8 and 1.92 nm respectively. The total surface area and hydrophobic content increase from 72 to 92 nm^2^ and from 5.6 to 7.4 nm^2^, respectively. The packing quality of the structure steadily declines: S-values after 5 and 10 μs of MD simulation are c.a. 100 and 77, respectively. Deviation of the latter model from that in water at normal temperature is shown in Fig. S2 in terms of the differential 3D_1D plot (blue curve).

The most prominent changes of the viscumin structure are observed in CHCl_3_/MeOH mixture. In this case, final RMSD and gyration radius reach 1.5 and 2.2 nm, respectively, while the total and the hydrophobic surfaces increase to 107 and 14 nm^2^. Interestingly, unlike steady degradation (observed in urea), the packing quality rapidly drops and after 5 μs of MD simulation remains almost constant - *S*-values after 4 and 10 μs of MD run are 27.31 and 27.70, respectively. Moreover, the corresponding 3D_1D profiles are rather similar (data not shown). Final deviation of the CHCl_3_/MeOH-adapted model from that in water at normal temperature is shown in Fig. S2 in terms of the differential 3D_1D plot (green curve). Analysis of the latter leads to the following two conclusions: (1) Both models become much less suitable for water environment – corresponding values of differential 3D_1D score are almost exclusively positive; (ii) Protein reorganization pathways in the two solvents are drastically different. In more detail, this will be discussed below. To get further insight into the conformational behavior of MLA in water (at normal and elevated temperature) and in two non-aqueous media, detailed analysis of the corresponding MD trajectories was carried out.

**Figure 2:**
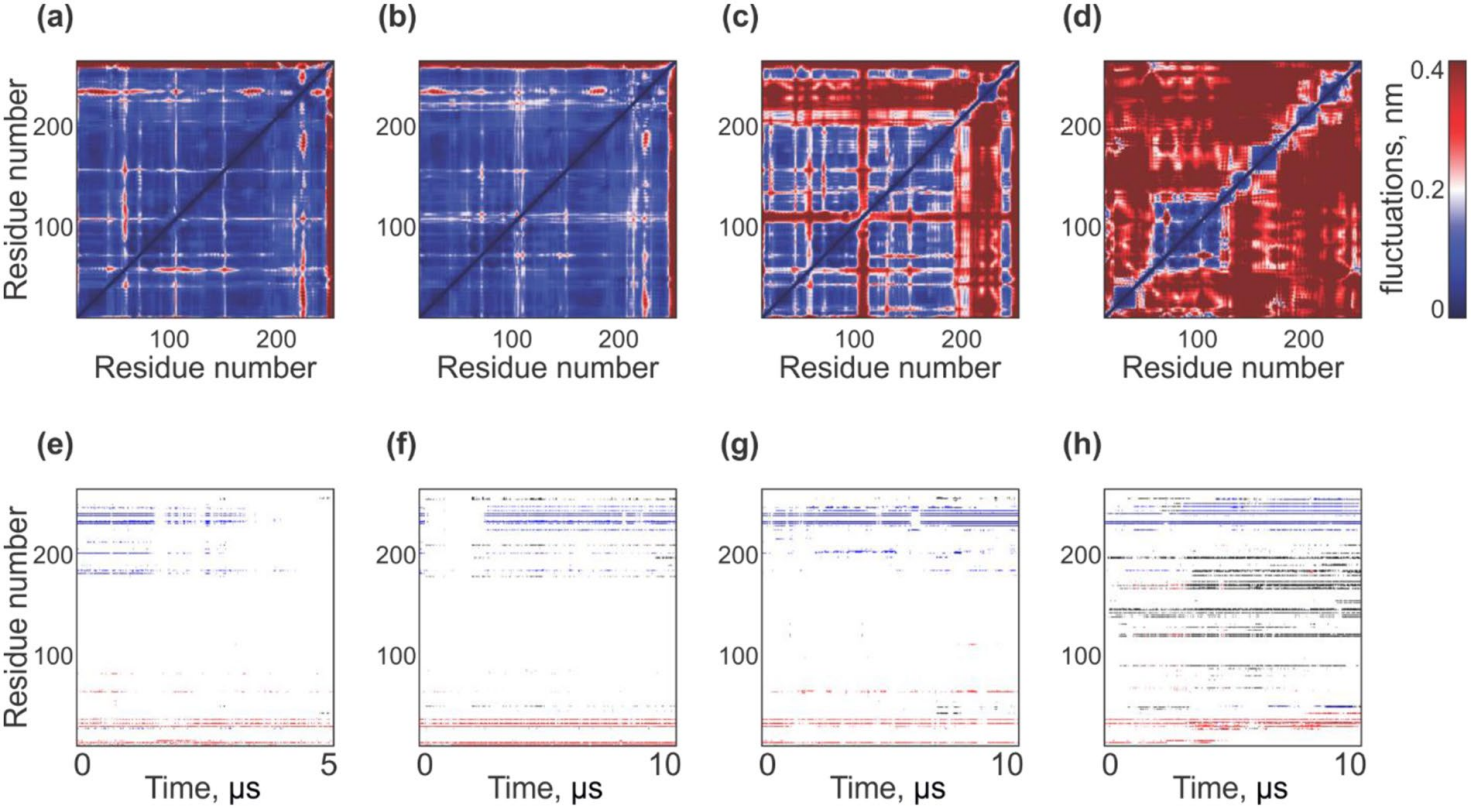
Solvent and temperature effects on the structure and dynamics of MLA as probed by MD simulations. Upper panel: Fluctuation maps of MLA in water at 310 K (**a**) and 340 K (**b**), in urea (**c**), and in CHCl_3_/MeOH mixture (**d**). Blue and red regions correspond to mutually conserved and fluctuating residues. Bottom panel: Maps of participation of protein residues in formation of surface hydrophobic clusters. N- and C-terminal hydrophobic clusters are colored red and blue, respectively. The others are shown in gray.

In the course of MD run in water at 310 K, the secondary structure of MLA remained generally stable (see Fig. S3a). The main changes were related to partial destabilization of the helices h6 (163–169), h9 (234–236), and the N-terminal part of h8 (190–200). In addition, residues in the region 96–100 occasionally formed a π-helix. Inspection of the residue-residue fluctuation map (Fig. 2a) shows that the main structural rearrangements occur in the C-terminal part of the protein (residues 246–254) at the region of the new helix appearance and close to residue 50. Only two hydrophobic clusters were located on the protein surface (Fig. 2e). The first (the N-terminal cluster shown as red on the maps) includes the residues L23, F27 (helix h1) and L4 (β-sheet A). This cluster exists during the entire simulation time and has the average surface area (*S*_*m*_) equal to 0.7 nm^2^. The second cluster (Interfacial cluster, blue) is located close to the β-sheet C (213–232). The hydrophobic cluster at this location usually includes one or two residues from the β-sheet C and one of the neighboring residues (W174 (h7) or Y191 (h8)). It has *S*_*m*_ = 0.8 nm^2^. This cluster becomes weaker when fluctuations of the helix h9 are observed (at the end of MD). These changes can be explained by relaxation of the A/B subunit interface - the hydrophobic residues tend to be hidden from water.

**Figure 3:**
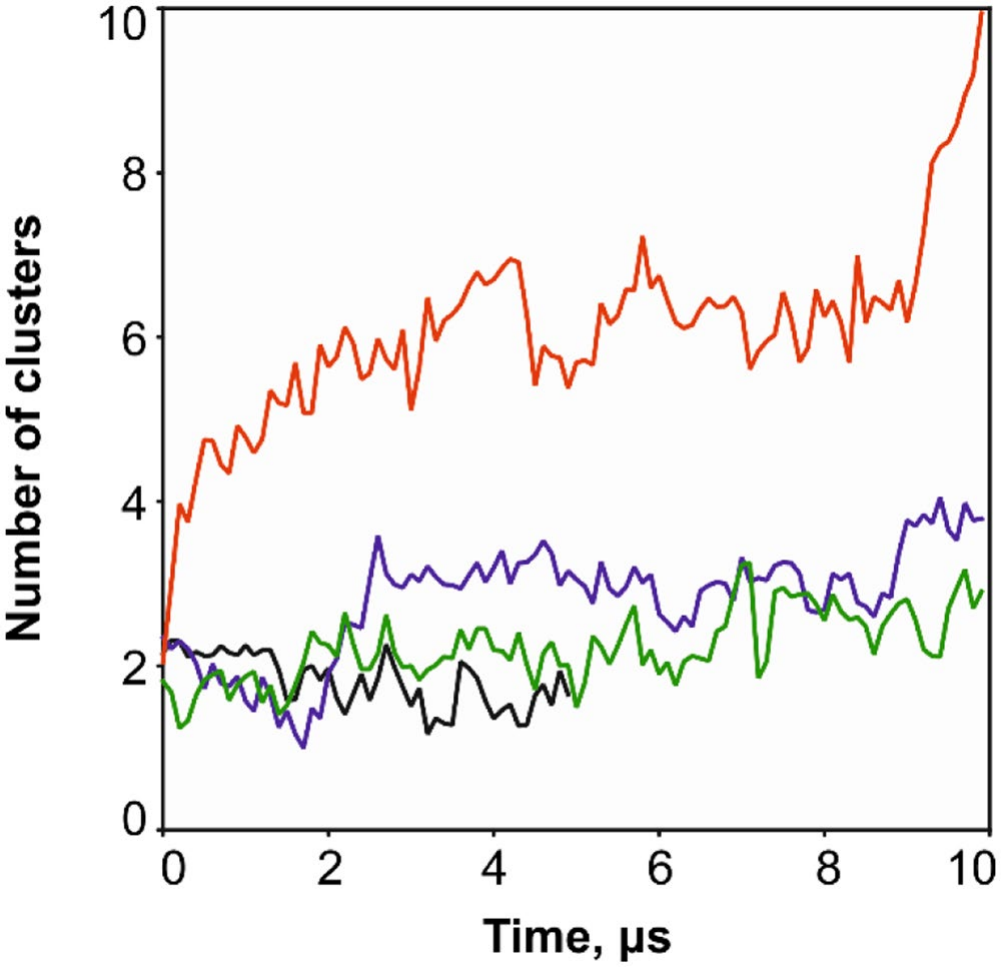
Time-dependent evolution of the number of hydrophobic clusters on the surface of MLA. Results of MD simulations in water at 310 K (black) and 340 K (blue), in urea (green) and in CHCl_3_/MeOH mixture (red).

Increasing the temperature to 340 K does not lead to noticeable destabilization of the MLA secondary structure (Fig. S3b) and fluctuations observed at 310 K occur also at 340 K. In addition, formation of two π-helices in the regions 46–50 and 213–216 was detected. Analysis of the residue-residue fluctuations map (Fig. 2b) shows that in this case the structural rearrangements were similar to those observed at 310 K. The hydrophobic clusters were also unchanged (Fig. 2f). As in MD at 310 K, the N-terminal cluster exists throughout the simulation time and has *S*_*m*_ = 0.6 nm^2^. However, at the elevated temperature, the interfacial cluster becomes more prominent: it has *S*_*m*_ = 0.6 nm^2^ and is observed right up to the end of the MD simulations. As seen in Fig. 3, at lower temperature, the number of hydrophobic clusters decreased with time, and *vice versa* - at 340 K, the C-terminal cluster increases at the end of the simulation. It is proposed that at 340 K relaxation of the A/B subunits interface does not occur and the hydrophobic cluster lying on it can be a potential future site of membrane binding.

As mentioned above, MLA in urea demonstrated large conformational changes resulting in a rather less compact structure than in aqueous solution. At the same time, its secondary structure did not change significantly (Fig. S3c). The only prominent difference was observed for the helix h8 (189–209) which was less stable in urea than in water. In addition, the C-terminal helix h9 disappeared. Analysis of the fluctuation map (Fig. 2c) showed that the structural rearrangements delineated in water became stronger in urea. Also, some perturbations of the helix h8 (189–209) and termini of the β-strand A (50,100) were found. Partial unfolding of viscumin proceeded as follows: initially, the α-helix h8 was melting thus resulting in a large intramolecular mobility of the β-sheet C. Surface hydrophobic clusters were the same as in water (Fig. 2g). Population of the N-terminal cluster was only about 40%. In addition to residues L4, L23, F27, it included residues from other regions, which changed their location in MD (F53, L55, F101). The mean surface area of this cluster (*S*_*m*_) was 0.5 nm^2^, whereas that of the interfacial cluster was 0.8 nm^2^. The latter was observed over the entire simulation time and included additional residues: W174, L187, Y191, M192, L193, L195 (helix h8), F214, I218, L220, L222, V228, L230, I233, V236, I237 (β-sheet C). In this case, the cluster contained residues from the hydrophobic side of the β-sheet C and several residues from the molten region h8. Analysis of the time evolution of the quality of the structure (3D_1D score) shows that during the first 5 μs of dynamics, the largest perturbations occur in the regions 35–58, 98–103, 172–182, and 222–238 (Fig. S4), while during the next 5-μs interval, residues involved include 35–58, 98–103, 190–200 and more importantly the C-terminal residues 210–220 and 225–250.

In the chloroform-methanol environment, the protein structure demonstrated the largest perturbations in comparison with the water-adapted conformation. Such rearrangements are not a usual feature of denaturation which in this case is also accompanied with degradation of the residue packing and spatial structure. Apart from the overall swelling and turning inside out (see above), the secondary structure of MLA was well preserved (Fig. S3d). In the beginning of the simulation, the helices h6 and h7 merged. Then, the helix h7 underwent reversible transitions to a π-helix. Stability of the helix h8 (189–209) was similar to that observed in water. The size of the helix h9 (234–236) was also increased to 235–240. Analysis of the fluctuation map (Fig. 2d) showed that in the CHCl_3_/MeOH medium, the protein lost many long-distance contacts required for maintenance of its globular shape. As a result, stable dynamic domains corresponding to the initial secondary structure elements, which move independently during MD, were detected.

The most important conformational transformation of MLA in the membrane-like environment is related to exposure of the hydrophobic protein regions, which were initially buried inside the globule. As seen in Fig. 3, up to 10 hydrophobic domains (clusters) were detected on the protein surface. Most of them correspond to nonpolar sections of helices, which originally participated in intramolecular contacts in water. Time-dependent formation of these clusters is shown in Fig. 2h. Based on this information and on the data of contacts between the secondary structure elements, rearrangement of MLA can be divided into several stages. Apart from the presence of hydrophobic clusters, which are also observed in water and urea, interactions of secondary structures degrade in the CHCl_3_/MeOH medium, thus leading to the appearance of large hydrophobic zones on the protein surface. At the first stage (up to 0.2 μs), contacts between the helices h4 (131–144) and h5 (148–163) are weakening. Then (up to 3.5 μs), the same event occurs for the helices h1 (13–28) and h7 (169–183). In addition, tight packing of the β-sheets A and B is progressively disturbed. Such a degradation of the contacts between the secondary structural elements results in two effects. Firstly, the protein tends to adopt an extended conformation with a large number of hydrophobic regions on the surface. Secondly, these regions move independently and are capable of adapting to the membrane binding. This also implies that the protein dynamics is considerably different compared to that observed for the globular structure. The above picture is completed with the analysis of 3D_1D scores of the conformations extracted after 5 and 10 μs of MD. As indicated above, the major perturbations occur already on the first time interval – the structure quality seriously falls in numerous regions (1–11,14–28, 35–41, 50–76, 108–121, 129–150, 152–167, 169–186, 188–195, 211–250, Fig. S5).

Representative MD states obtained at the end of the simulations in all three solvents were further tested on their ability to interact with an implicit membrane model – so-called “hydrophobic slab”. This was done using MC simulations in dihedral angle space. The results obtained are presented below.

### MC simulations of native and refolded protein in the presence of implicit membrane

**Figure 4:**
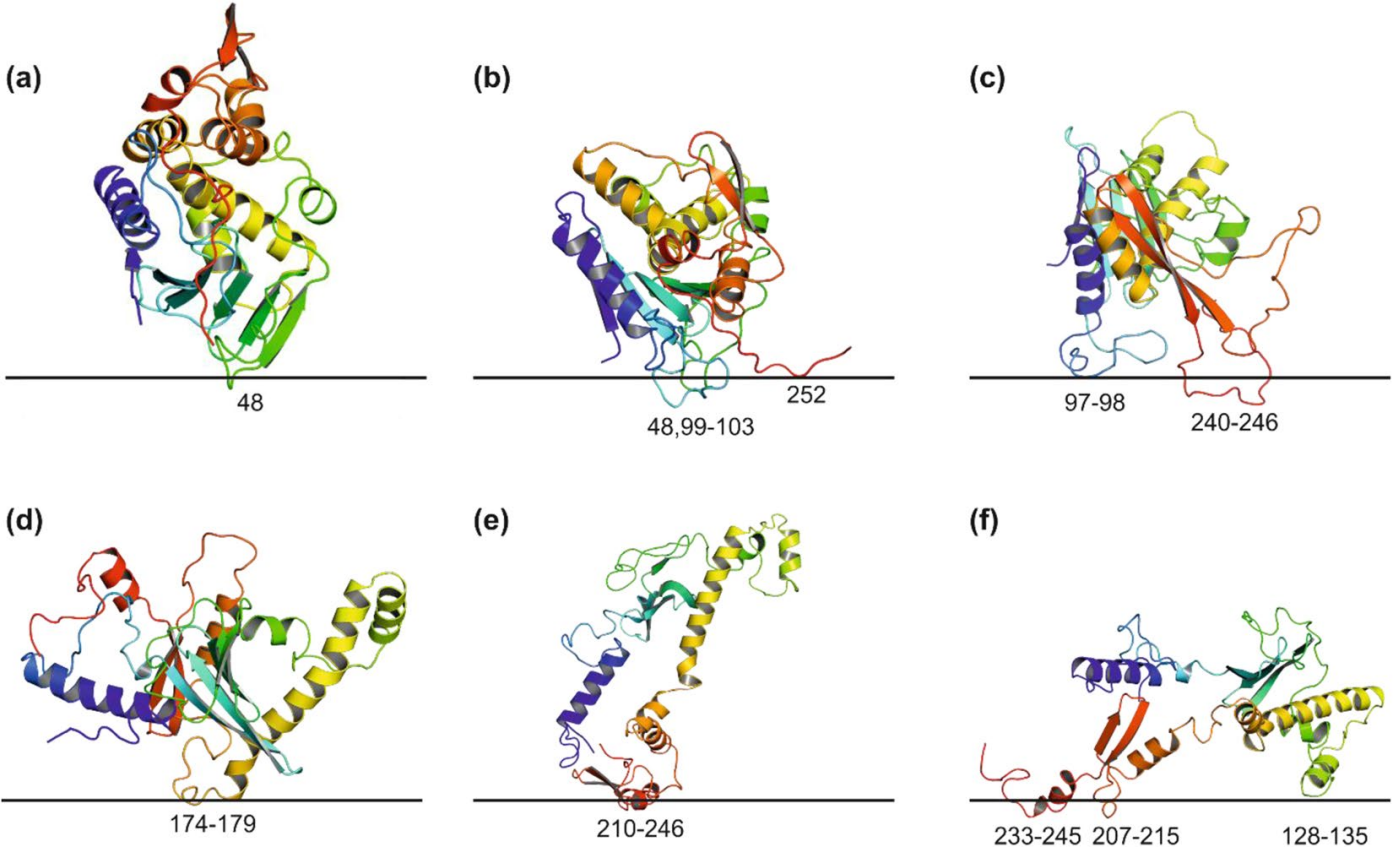
Results of MC simulations of the water-(**a**), urea- (**b**,**c**) and CHCl_3_/MeOH-adapted (**d**,**e**,f) models of MLA in membrane-mimic hydrophobic slab. Disposition of the most-populated low-energy states with respect to the membrane surface (shown with horizontal line). The starting structures correspond to the states obtained after 5 μs (a) of MD simulation in water; 5 μs (b) and 10 μs (**c**) of MD simulation in urea, 4 μs (**d**) and 10 μs (**e**,**f**) of MD simulation in CHCl_3_/MeOH. Membrane-binding sites are indicated with residue numbers. Protein is shown in a ribbon presentation.

Disposition of the observed lowest-energy states of MLA with respect to the membrane surface (indicated with a horizontal line) is shown in Fig. 4. It is clearly seen that among the energetically favorable conformations of MLA (with the total energy *E* ≤ *E*_*min*._ + 15 kcal/mol, where *E*_*min*._ is the minimal total energy) there are states contacting the membrane, while the starting structures were always placed in the polar phase, outside the hydrophobic slab. At the same time, protein binding to membrane critically depends on the environment, which was used to generate the starting structures. Below, this issue is considered in more detail for each of the solvent-adapted forms of MLA.

### Water-adapted structure of MLA

**Figure 5:**
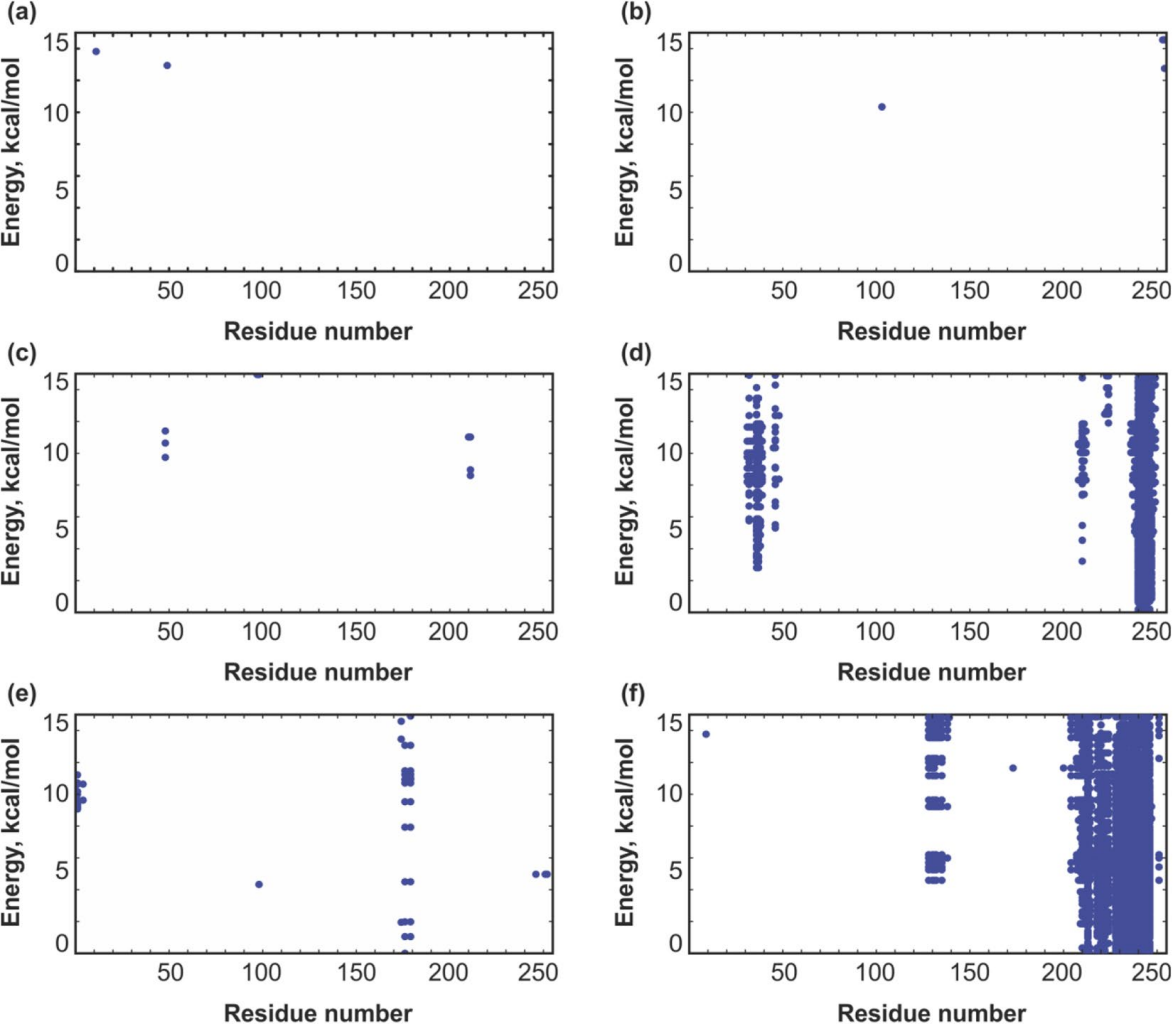
Results of MC simulations of different solvent-adapted models of MLA in the presence of the membrane-mimic hydrophobic slab. Contacts of residues with membrane in the lowest-energy MC-states are shown with blue dots. X-axis: residue number; Y-axis: total energy of the system. Contacts maps are presented for the structures obtained after MD relaxation in water at 310 K (**a**) and 340 K (**b**); after 5 and 10 μs MD in urea (**c**,**d**); after 4 and 10 μs MD in CHCl_3_/MeOH mixture (**e**,**f**). In each case, the lowest-energy of the found MC state was assigned to zero.

As seen in Fig. 5a,b, these two protein models (obtained at 310 and 340 K) prefer to stay in the polar environment and reveal very few contacts with the membrane. The membrane-associated states are rather poorly populated – their total energies are 10–12 kcal/mol higher than *E*_*min*_. Possible membrane-binding sites (with the energies within 15 kcal/mol from *E*_*min*._) are formed by residues 11, 48 (Fig. 5a) for the structure adapted to water at 310 K and 103, 252–254 (Fig. 5b) for the structure adapted to water at 340 K. Noteworthy, residues 11, 48, and 103 are located close to the N-terminal hydrophobic cluster on the MLA surface. Thus, it is reasonable to conclude that interaction of the water-adapted MLA with the membrane is rather weak and occurs through the N-terminal hydrophobic cluster. Membrane contacts through the C-terminal residues are observed only at the elevated temperature (340 K) – the corresponding MD-structure is somewhat distorted in this region, which in the native protein represents an interface with the B-subunit of MLA (see above).

### Urea-adapted structure of MLA

The structure obtained after a 5 µs MD run in urea (Fig. 5c) also reveals rather weak contacts with the membrane-mimicking hydrophobic slab – *via* the following residues: 210–211, 48, 97–98. Hereinafter, the segments are listed by ascending total energy values. It is of interest to note that among such states, the most energetically favorable ones (with membrane-bound residues 210–211) lose some 8 kcal/mol to the *E*_*min*._- conformation, which remains in the water-mimicking medium (Fig. 5c) and does not interact with the slab. However, despite very few favorable contacts with the membrane, this structure demonstrates stronger binding as compared with the previously mentioned water-adapted models. A subsequent 5 µs of MD-simulated reorganization of MLA in urea resulted in considerable strengthening of protein-membrane interactions. Thus, the structure taken after 10-µs MD demonstrates much more prominent contacts with the hydrophobic slab (Fig. 5d). In contrast to the previous case, the most energetically favorable states are adsorbed on the membrane surface with the C-terminus - residues 240–246. The second binding region – including residues 31–39 and 46 – appears in the states separated from *E*_*min*._ by ~3–4 kcal/mol. Together with the C-terminal region, these residues form a kind of two-site hydrophobic motif anchoring MLA in the membrane. As seen in Fig. 4c, the loop 240–246 (red) deeply penetrates into the hydrophobic zone, while the segment 31–39 (blue) just “leans” on the membrane surface.

### Chloroform/Methanol-adapted structure of MLA

As in the case of urea, two structurally different models of MLA were taken from MD trajectory in this mixed organic medium, which mimics well the membrane environment. As shown above, in the course of MD simulations in explicit CHCl_3_/MeOH solution, the initial water X-ray model of MLA undergoes global structural rearrangements accompanied with exposure of nonpolar protein core regions to solvent. Accommodation of the two MD-derived models – after 4 and 10 µs of simulations – with respect to the implicit membrane is shown in Fig. 4(d–f). For the corresponding MC-states, total energies and residues in contact with the membrane are given in Fig. 5e,f. It is evident that both models demonstrate much stronger interactions with the hydrophobic slab as compared with the urea-adapted forms. Already after first 4 µs of dynamics, at least four protein regions inserted into the membrane were found among the low-energy MC states. These are residues 174–179, 98, 246–252, and 1–4. Protein embedding becomes very favorable at the end of the MD run (near 10 µs) - energies of numerous MC-states fall in the interval [*E*_*min*._ + 15 kcal/mol]. In these states, MLA binds to the slab with the segments 207–214, 218–220, 228–246, 251, 128–135, 9, 173, which form two main membrane binding sites – residues 210–215, 218–246 and 128–135, 207–215, 233–245, 251 (Fig. 5e,f). These regions are partially overlapped by the C-terminal segments 210–215, 233–246, while the specific determinants are 218–232 and 128–135/251, respectively. Adsorption *via* the first site (Fig. 5f) is accompanied with deep insertion of the extended MLA region into the hydrophobic zone, while the alternative binding mode (Fig. 5e) is characterized by immersion of much shorter fragments.

## Discussion

The principal discovery of this work is that the well-packed water soluble globular protein MLA undergoes global conformational reorganization in the membrane-mimic medium in such a way that its spatial structure becomes well-suited for interaction with the model cell membrane. It is important to outline that such a result was not obvious until the computational experiments had been carried out. Possible reasons for this uncertainty are: (1) In the case of “usual” refolding/denaturation (as in commonly used solvents, e.g., in urea or under elevated temperature), structural rearrangements of the protein can be very different from those observed in the membrane mimic; (2) In organic solvent the initial globule enriched with secondary structure elements could just begin to disintegrate by losing interactions between them and unravelling of the regular structures; (3) The protein structure distorted by the organic solvent can be unsuitable for membrane binding.

How far are these results consistent with the available experimental data? The latter were obtained mainly on ricin - a close homolog of viscumin. It has been shown that (i) the A chain of ricin (RTA) is capable of embedding into the cell membrane; (ii) it undergoes significant structural rearrangements upon the membrane binding. Two sets of experimental measurements concerning ricin-membrane interactions were obtained. In the first, the ability of RTA with respect to membrane binding was investigated using detergent extraction, gel filtration chromatography, sedimentation analysis, fluorescence spectroscopy and circular dichroism^20,23^. Based on mutagenesis data it was shown that isolated RTA interacts *via* its C-terminal hydrophobic region V245-V256 with lipid membranes and partially unfolds in the process^26^. The accessibility of particular regions and individual residues to a lipid bilayer was characterized at 20 °C and 37 °C^20^. It was shown that at 20 °C, the following RTA residues are accessible to the membrane: E61, Q98, R114, Q128, E135 (corresponding residues in MLA are: E57, P93, R106, R120, D126), while at 37 °C, new membrane-embedded sites appear – R31 (Y27 in MLA), I249 (I237), C259 (C247). At the same time, simply increasing the temperature is not sufficient to induce membrane translocation of MLA^27^. This coincides well with our MD-based conclusion that protein structural rearrangements in water at 340 K do not promote strong binding to the model membrane.

The second set of data represents the results of RTA interaction with neutralizing antibodies, which are supposed to interfere with the penetration of toxin through the membrane by blocking the unfolding of RTA^28^. The corresponding regions in MLA are R94-F101, T151-M162, and R175-F186. In addition, β-sheet 3 (218–222, 225–230) is an epitope for non-neutralizing antibodies, so it is interesting to access its behavior upon protein rearrangement on the membrane. In addition, introduction of a disulfide bond Cys251-Cys255 into RTA by site-directed mutagenesis did not affect *in vitro* catalytic activity and RTB binding, but resulted in about 15-fold less cytotoxicity^22^. Therefore, it was assumed that stabilizing bond Cys251-Cys255 prevents unfolding of RTA, which is necessary for membrane translocation. In summary: existing experimental data on mapping with antibodies indicate that the main conformational changes during interaction with the membrane apparently occur with β-sheet 3 and helices h7 and h8 of the A-chain. At the same time, one should bear in mind that the molecular mechanisms determining the observed effects of both, neutralizing and non-neutralizing antibodies, are not fully understood. Therefore, they still cannot provide direct structural information for the behavior of the toxin on translocation.

In summary, the results of the simulations described here agree well with experimental observations especially when taking into account the close homology of RTA and MLA. Firstly, the globular MLA is rather stable in water at 310 K – the main changes in the structure occur close to the interface between subunits A and B. During MD relaxation of this interface was observed. At an elevated temperature (340 K), MLA undergoes partial destabilization – but the hydrophobic cluster on the A/B interface exists over all simulation time. In urea, a further increase in the size of this cluster was observed and this was shown to be due to melting of the helix h8, rearrangements of the β-sheet 3 and structural changes in the h9 and C-terminal residues. For the structures obtained from this method of degradation, interactions with the hydrophobic slab are implemented by residues whose importance for membrane binding has been shown experimentally. These are the C-terminal β-sheet 3, helix h9, and the regions 246–254, 94–101. The former two correspond to the A/B interface of the toxin. The nonpolar stretch along with the site 246–254 was identified as a potential, but weak, membrane binder. Similar conclusions were made for RTA at 20 °C based on experimental measurements^20^.

In the CHCl_3_/MeOH mixture, MLA refolded in another way. In this solvent, the protein structure is practically turned inside out while still effectively preserving the secondary structure. This very large conformational rearrangement probably occurs mainly upon the incorporation of MLA into the membrane. On the other hand, it may be possible to find antibodies stabilizing the structure of MLA in those regions which were first to change structurally. According to the first stage in the molecular dynamics simulations, weakening of contacts between the helices h4(131–144) and h5(148–163) was observed. According to Legler, the region T151-M162 is one of the sites of interaction with neutralizing antibodies blocking the unfolding of RTA necessary for membrane translocation^28^. In addition, unfolding of the A chain was experimentally demonstrated both for RTA and MLA^17,18^. In the cell, the unfolded A-chain is then misrouted to the ER-associated degradation pathway and translocated into the cytosol^18^. The membrane-embedding MLA region predicted by computer modelling, includes amino acid residues 207–215, 218–220, and 228–246. This corresponds well with the region of RTA which is most likely involved in ER membrane binding. It is worth noting that the importance of the C-terminal region of RIPs was independently confirmed in experiments with the A-chain of pulchellin – close homolog of ricin and viscumin^29^. Taken together this behavior and the identified protein regions coincide well with the experimental observations^20,22,26,29^. The computational results reported here, permit prediction of several new, not yet experimentally tested as shown above, MLA-based peptides, which can be further used as linear epitopes to produce antibodies, which can specifically recognize membrane-reshaped forms of the protein. The most promising of these are: 6–9, 37–40, 53–58, 128–135. Under normal conditions in water, these determinants are buried inside the globule, and become exposed only with membrane-assisted refolding. As shown above, some of these (6–9, 128–135) are critical for membrane binding, while the others (37–40, 53–58) can be considered just as markers of the “membrane adapted” states. It is important to note that the structural rearrangement pathways may differ significantly depending on the environment and the temperature. Thus, MLA conformational changes in the membrane mimic (CHCl_3_/MeOH) do not resemble those in urea. In addition, thermal destabilization of MLA in water proceeds in a specific way. Depending on the MD simulation conditions, the toxin’s conformational rearrangements and the membrane-binding ability increase in the sequence: water (310 K) < water (340 K) < urea (340 K) < CHCl_3_/MeOH (340 K). We should also note that the MD results were reproduced in several independent simulations (see Methods) thus strengthening the conclusions.

Obviously, the reported computational view gives a rather rough picture and just “catches” a general tendency of a membrane-induced restructurisation of MLA. To assemble the puzzle, more details are needed, such as the structural/dynamic behavior of the toxin at the water-bilayer interface, the role of anionic lipids (which were shown to be important for RTA)^23^ and temperature^20^. Deciphering of the details of the regulatory mechanisms of viscumin and related toxins (ricin, Shiga and Cholera toxins) at the atomic level with respect to unfolding and subsequent membrane translocation are of paramount importance because this opens up avenues for the design of new molecules as modulators of the toxins’ activity.

## Materials and Methods

### Molecular dynamics

The spatial structure of the chain A of viscumin was extracted from PDB entry 2RG9 and five C-terminal residues 249–253 (UniProt ID: P81446)^30^ were added using the Modeller 8.2 software^31^. Molecular dynamics (MD) simulations were performed with the GROMACS package^32^ version 5.1.2, compiled with CUDA GPU support, and the improved model of the all-atom AMBER99SB-ILDN force field^33^. An SPC water model was used^34^. Topology and force field parameters for chloroform, methanol, and perchlorate ion were generated using the ACPYPE^35^ software package based on Ambertools^36^ version 1.5. Correctness of the force field parameters for the latter was specially checked via pure liquid MD simulations at temperatures 303, 313, 323, and 343 K. Analysis of the equilibrated systems revealed that the calculated macroscopic averages are in good agreement with experiments. For instance, average densities deviated by less than 5% from the experimental data. MD simulations were carried out with a 2 fs time step and imposed 3D periodic boundary conditions in a dodecahedron box. The initial size of the box was 8.9 nm, which corresponds to the box volume 500 nm^3^ and minimal distance from solute to the box edges was 1.4 nm. Protein was placed in the center of a pre-equilibrated solution box, overlapped solvent molecules were removed and then some solvent molecules were substituted as necessary by ions. The total composition of the simulated systems was: (MLA in water) protein, 14962 waters, 4 Na^+^; (MLA in CHCl_3_/MeOH) protein, 1855 CHCl_3_ molecules, 1940 MeOH molecules, 10 Li^+^, 10 ClO4^−^, 14 Na^+^, 10 Cl^−^; (MLA in urea) protein, 2400 urea molecules, 8139 waters, 4 Na^+^. In the latter case, aqueous 8 M urea solution was taken before addition of MLA. The systems were equilibrated by steepest descent energy minimization followed by heating from 5 K to 340 K (310 K) during a 1 ns MD run. Finally, long production MD runs (5 μs for water at 310 K, 10 μs for water, CHCl_3_/MeOH, and urea - all at 340 K) were carried out for each system.

Molecular dynamics was performed using a leap-frog integrator^37^. A Verlet cutoff scheme was used with the same cutoff values for van der Waals and electrostatic interactions. The latter were treated using the particle-mesh Ewald summation with fourth-order spline interpolation^38^. An initial cutoff value of 1.2 nm and Ewald grid spacing of 0.12 nm were tuned during the calculations to balance CPU-GPU loading. MD simulations were carried out using the isothermal-isobaric (NPT) ensemble with an isotropic pressure of 1 bar and a constant temperature of 340 K (One MD trajectory in water was obtained at 310 K). The pressure and the temperature were controlled using the V-rescale thermostat^39^ and Parrinello-Rahman barostat^40^ with 1.0 and 0.1 ps relaxation parameters, respectively, and a compressibility of 4.5 × 10^−5^ bar^−1^ for the barostat. In order to check reproducibility of MD results, several shorter independent simulations were carried out: two 100 ns MD runs in water at 310 K; two 100 ns simulations in 8 M urea at 310 K and two 100 ns MD runs at 340 K; one 4 μs run in CHCl_3_/MeOH at 340 K.

In order to increase the stability of the dynamics, the bonds with hydrogens were transformed into constraints, which were calculated using the fourth-order P-LINCS algorithm^41^.

### Fluctuation maps

For a given time interval of MD, analysis of protein fluctuations was performed using the following approach. For each MD frame, the matrix of distances between CA atoms of residues was calculated. Fluctuation of these distances over a given period of MD represents a mutual mobility of the protein regions. That is why, the standard deviations of these distances (referred to as “fluctuation value”) were evaluated during a given time period. The resulting matrix is presented in the form of a ‘fluctuation map’. The fluctuation value correlates with mutual mobility of the atoms. Low values (<0.2 nm, blue regions on the map) indicate that these protein fragments form a single dynamic domain, whereas their high values (>0.3 nm, red regions) point out their independent movement.

### Surface analysis

Dot Connolly surfaces of MLA were calculated with a probe radius 0.14 nm (corresponding to water) and dot density 3000 points/nm^2^ using in-house software. In each point, a value of the molecular hydrophobicity potential (MHP) induced by protein atoms was calculated as described elsewhere^42^. A point was treated as hydrophobic if the corresponding MHP value exceeded 0.3 a.u. (MHP is measured in logP units, where P is the distribution coefficient in a water/octanol-1 two-phase system.) To search the regions that can potentially interact with the membrane, analysis of hydrophobic clusters was carried out, determined using a grid-based clustering algorithm with cell size 0.3 × 0.3 × 0.3 nm^3^. To reduce the noise in the data, only large clusters (with surface area greater than 0.30 nm^2^) were analyzed. Hydrophobic clusters were characterized by their surface area, composition (MLA residues participating in cluster formation), and lifetime. The latter were calculated as follows. For each MD frame, hydrophobic clusters on the protein surface were delineated. If two clusters from consecutive frames have common residues, the lifetime of this cluster was increased in one time step.

### Monte Carlo simulations with implicit membrane model

MC simulations of several conformations of MLA - adapted to water (310 and 340 К), urea and CHCl_3_/MeOH media - were carried out using the two-phase implicit solvation model^43^. In the former case, the starting structure was that used in MD simulations in water (see above). For urea- and CHCl_3_/MeOH forms, the starting structures corresponded to one intermediate and the final configurations obtained *via* MD simulations in these solvents, as described above. The intermediate states were extracted from the corresponding MD trajectories after 5 and 4 µs. The protein accommodation near the membrane surface was explored *via* MC search in torsion angles space using the modified FANTOM program^44^. The starting structures were arbitrarily placed in the aqueous phase. The width of the interface region and the bilayer thickness were taken to be 0.15 and 3.00 nm, respectively. To change during the simulation orientation of protein with respect to the membrane, fragments of 21 dummy residues were attached to its N-terminus. The number of dummy residues is determined by the membrane half-width and the protein size. These “virtual” residues do not contribute to the energy of the system. The first atom of the N-terminal dummy residue was always placed in the center of the hydrophobic layer with coordinates (0, 0, 0). The protein models were considered in all-atom presentation. Ionization states of the residues were taken corresponding to pH 7.

All dihedral angles except the *ω* angles were sampled in “real” residues. Steps used in the variation of each dihedral were chosen randomly in the range of −180 to 180°. Nonbond interactions were truncated with a spherical cutoff of 1.4 nm. Electrostatic interactions were treated with a distance-dependent dielectric permeability *ε* = *4* × *r*. Before MC simulations, the structures were subjected to 100 cycles of conjugate gradients minimization. Acceptance of the MC-states was carried out according to the Metropolis criterion^45^. To cross the energy barriers between local minima, an adaptive-temperature schedule protocol was employed^44^. Several consecutive MC runs (c.a. 10^4^ steps each) with different seed numbers and sampled 3, 2, 1 randomly chosen dihedrals in the “dummy region” were performed without restraints. At each MC step, the structures were minimized *via* 50–100 conjugate gradients iterations. In each run, the initial conformation was the lowest-energy one found in previous runs. In total, ~3 × 10^4^ MC steps were performed for all proteins in one complete MC simulation.

Other details of the simulations (including inspection of the convergence problem in MC search) have been described elsewhere^40,46^. Analysis of the MLA orientations with respect to the membrane was carried out using auxiliary programs specially written for this. Resulting MC-states of MLA were analyzed using the following parameters: (i) total energy; (ii) residue disposition with respect to the membrane (position (*z*) of its center of mass along the slab normal – axis Z). Residue i was considered interacting with the membrane if |z_i_| < 1.5 nm.

### 3D_1D scores of the spatial models of MLA

The values of local (for each residue) and total 3D_1D scores were calculated as described elsewhere^25^. This was done using the programs generously provided by Prof. D. Eisenberg (UCLA).

## Electronic supplementary material

Supplementary information

## Data Availability

Datasets generated and/or analysed during the current study may be obtained upon request from the corresponding author.
