# Supplementary material for: Specific refolding pathway of viscumin A chain in membrane-like medium reveals a possible mechanism of toxin entry into cell
Source: Sci Rep. 2019 Jan 23;9:413. doi: 10.1038/s41598-018-36310-6 (PMC6344525; doi:10.1038/s41598-018-36310-6)
Supplement: Supplementary file 1 — Supplementary information [file 41598_2018_36310_MOESM1_ESM.docx]

**Specific refolding pathway of viscumin A chain in membrane-like medium reveals a possible mechanism of toxin entry into cell.**

Pavel E. Volynsky^1*^, Dmitry E. Nolde^1*^, Galina S Zakharova^2^, Rex A. Palmer^3^, Alexander G. Tonevitsky^2,4^, Roman G. Efremov^1,4**^

^1^ M.M. Shemyakin & Yu.A. Ovchinnikov Institute of Bioorganic Chemistry, Russian Academy of Sciences, Miklukho-Maklaya Street, 16/10, Moscow 117997.

^2^ Scientific and Research Center “BioClinicum”, Ugreshkaya Street, 2/85, Moscow 115088.

^3^ Department of Crystallography, Biochemical Sciences, Birkbeck College, Malet St, London WC1E7HX, UK

^4^ National Research University Higher School of Economics, Myasnitskaya ul. 20, 101000 Moscow, Russia

* Equal contribution.

** Corresponding author. Efremov Roman G.; Build. 16/10, Miklukho-Maklaya Street, Moscow, 117997, Russia; Phone: +7 903 743 16 56; E-mail: [efremov@nmr.ru](mailto:efremov@nmr.ru).

**
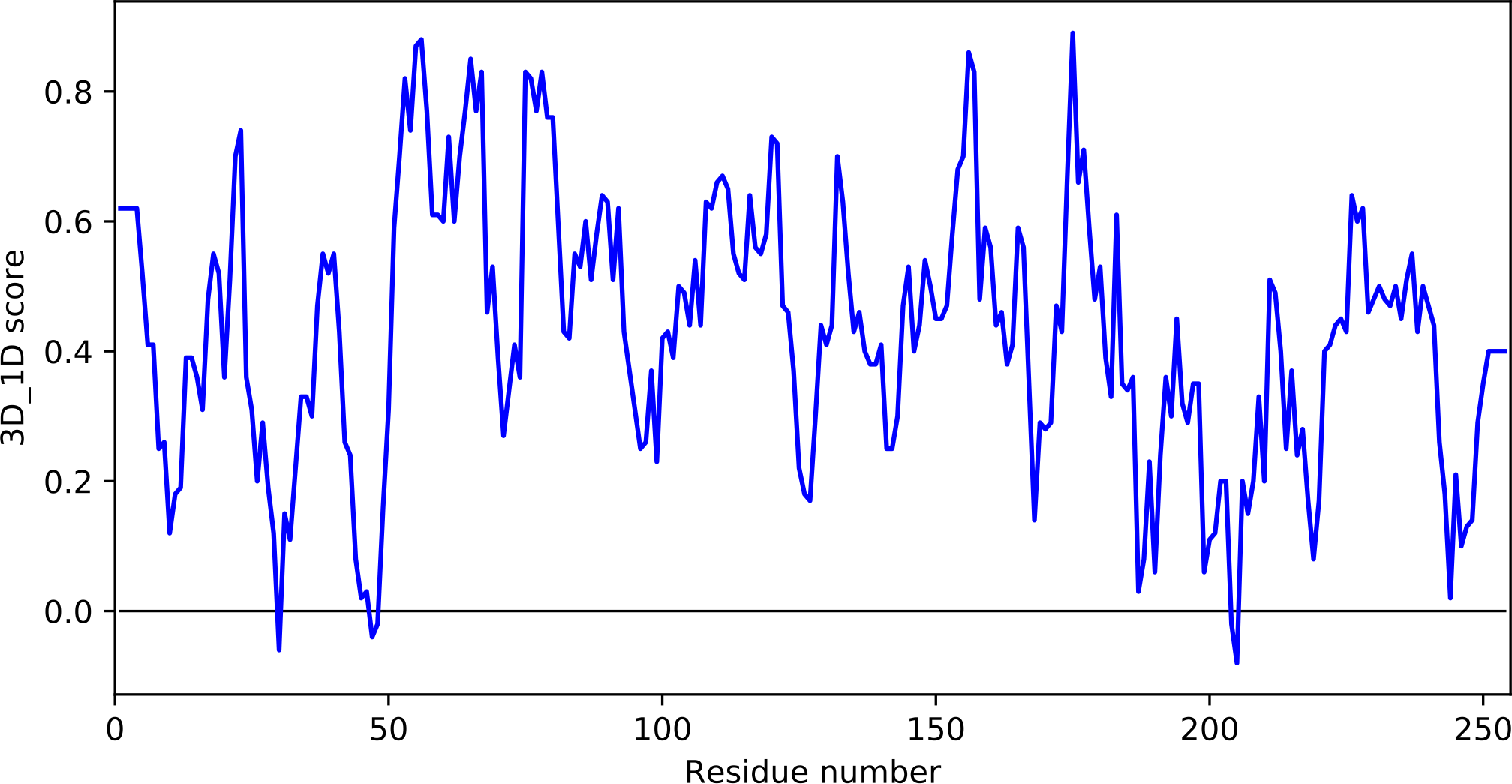
**

**Figure S1.** 3D_1D plot for water-adapted model of MLA after 5-μs MD simulations at T = 310 K.


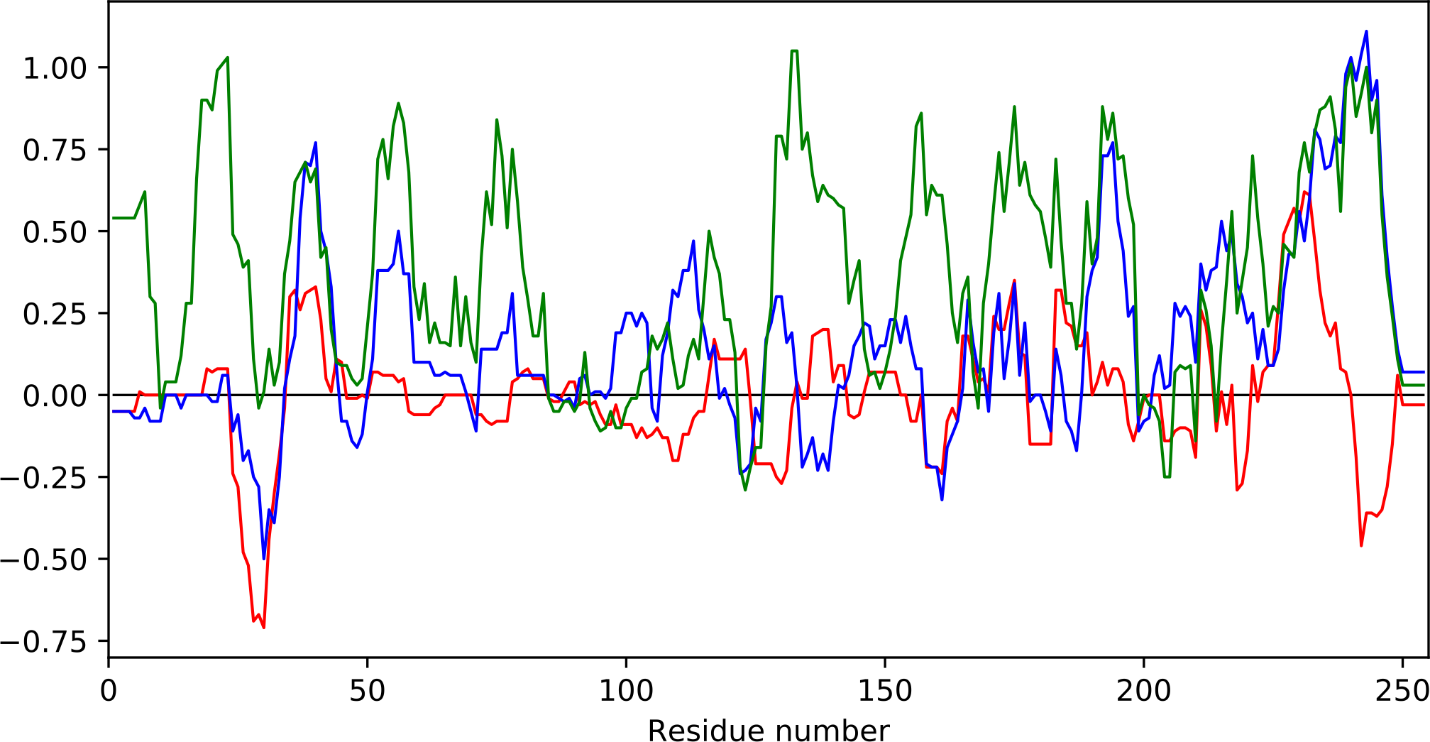


**Figure S2**. Structural reorganization of MLA in urea and CHCl_3_/MeOH mixture as revealed by 10-μs MD simulations at T = 340 K. Differential plots of the corresponding structural models obtained by subtraction of 3D_1D plots in water at 340 K (red), in urea (blue) and CHCl_3_/MeOH mixture (green) from the 3D_1D plot of water-adapted structure at T = 310 K. All structural MLA models, except the latter one, were obtained after 10-μs MD simulations (the latter - after 5-μs MD).


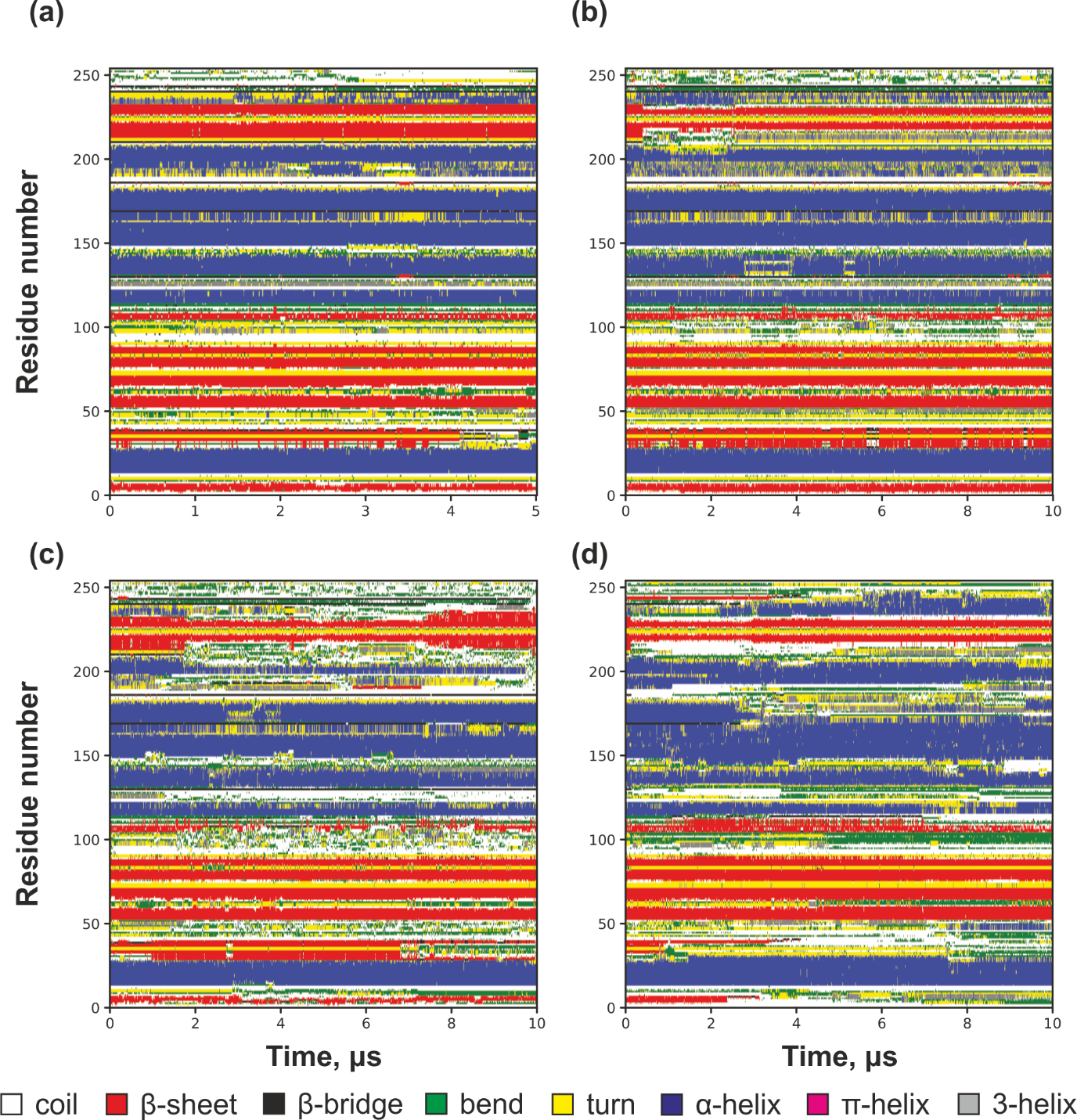


**Figure S3**. Time evolution of the secondary structure of MLA in the course of MD simulations in water at 310 K (a) and 340 K (b), in urea (c) and in CHCl_3_/MeOH mixture (d). The X-axis represents the MD trajectory time (in μs), while the residue numbers are shown on the Y-axis. Secondary structure elements are shown with different colors, as indicated in the bottom.


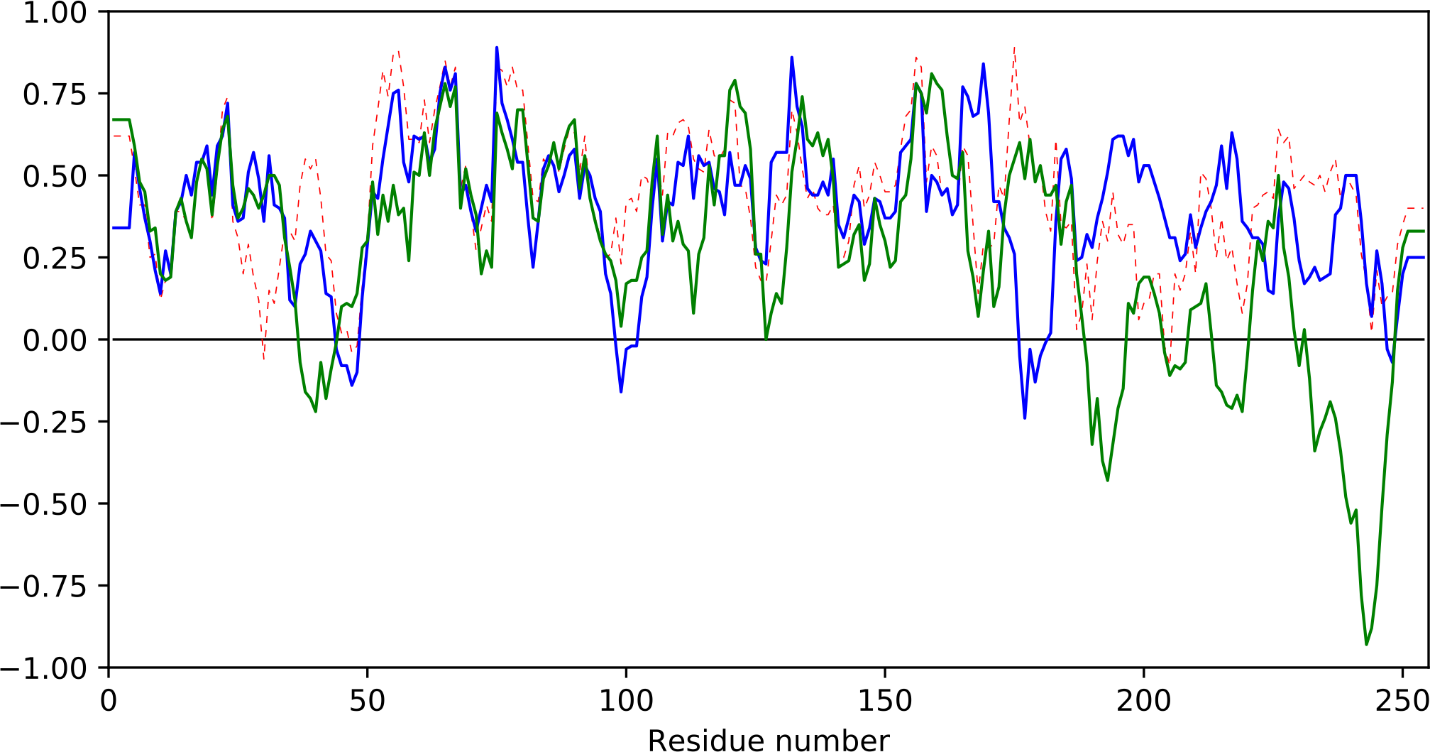


**Figure S4.** Time-dependent structural reorganization of MLA in urea. 3D_1D plots after 5-μs (blue) and 10-μs (b) MD simulations at T = 340 K. Red curve corresponds to the water-adapted MLA model obtained after 5-μs MD run at T = 310 K.


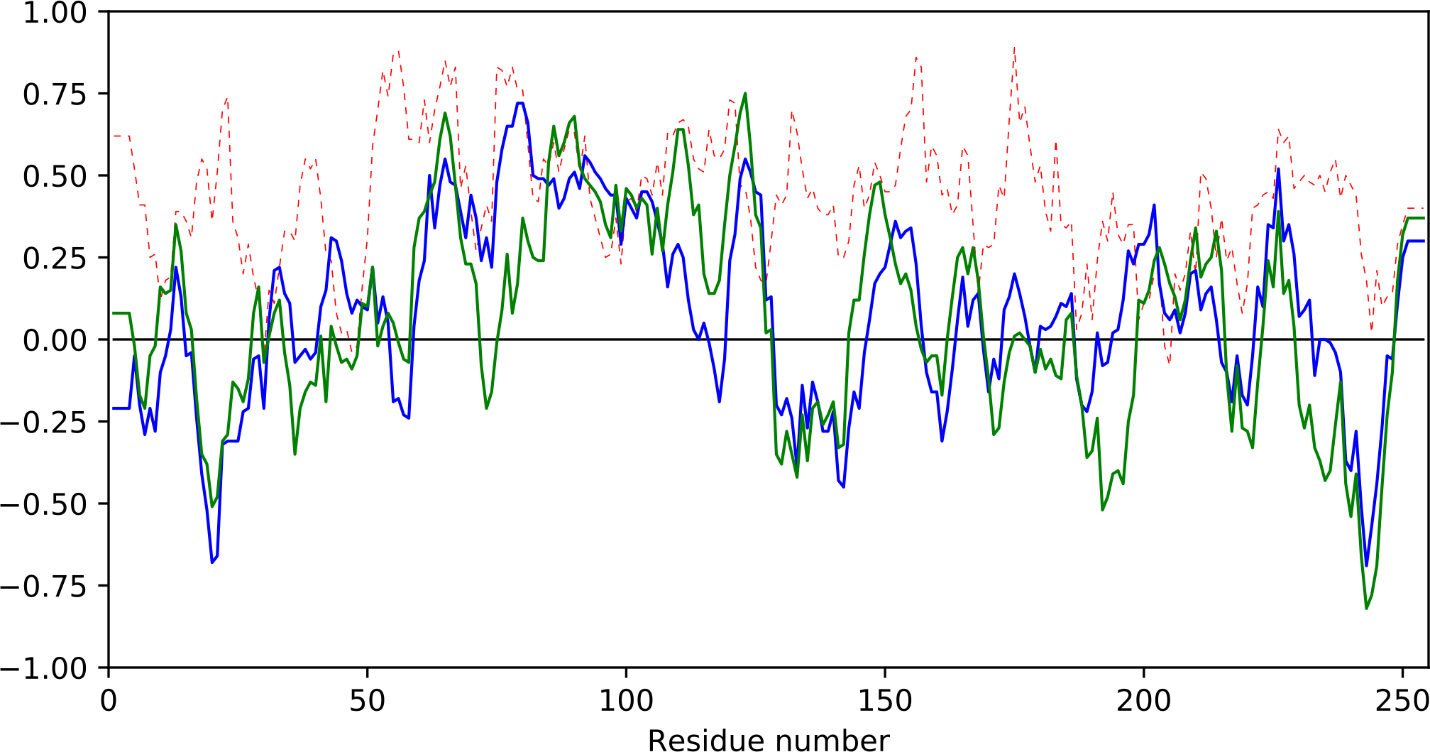


**Figure S5**. Time-dependent structural reorganization of MLA in CHCl_3_/MeOH mixture. 3D_1D plots after 4-μs (blue) and 10-μs (green) MD simulations at T = 340 K. Red curve corresponds to the water-adapted MLA model obtained after 5-μs MD run at T = 310 K.
